# Gene editing with ‘pencil’ rather than ‘scissors’ in human pluripotent stem cells

**DOI:** 10.1186/s13287-023-03394-5

**Published:** 2023-06-20

**Authors:** Ju-Chan Park, Mihn Jeong Park, Seung-Yeon Lee, Dayeon Kim, Keun-Tae Kim, Hyeon-Ki Jang, Hyuk-Jin Cha

**Affiliations:** 1 College of Pharmacy, Seoul National University, 1 Gwanak-ro, Gwanak-gu, 08826 Seoul, Republic of Korea; 2 Division of Chemical Engineering and Bioengineering, College of Art Culture and Engineering, Kangwon National University, Chuncheon, South Korea

**Keywords:** Human pluripotent stem cells, Disease modeling, Ex vivo therapy, Isogenic pair, Base editors, Prime editor, Base substitution, Cas9

## Abstract

Owing to the advances in genome editing technologies, research on human pluripotent stem cells (hPSCs) have recently undergone breakthroughs that enable precise alteration of desired nucleotide bases in hPSCs for the creation of isogenic disease models or for autologous ex vivo cell therapy. As pathogenic variants largely consist of point mutations, precise substitution of mutated bases in hPSCs allows researchers study disease mechanisms with “disease-in-a-dish” and provide functionally repaired cells to patients for cell therapy. To this end, in addition to utilizing the conventional homologous directed repair system in the knock-in strategy based on endonuclease activity of Cas9 (i.e., ‘scissors’ like gene editing), diverse toolkits for editing the desirable bases (i.e., ‘pencils’ like gene editing) that avoid the accidental insertion and deletion (indel) mutations as well as large harmful deletions have been developed. In this review, we summarize the recent progress in genome editing methodologies and employment of hPSCs for future translational applications.

## Background

**Fig. 1:**
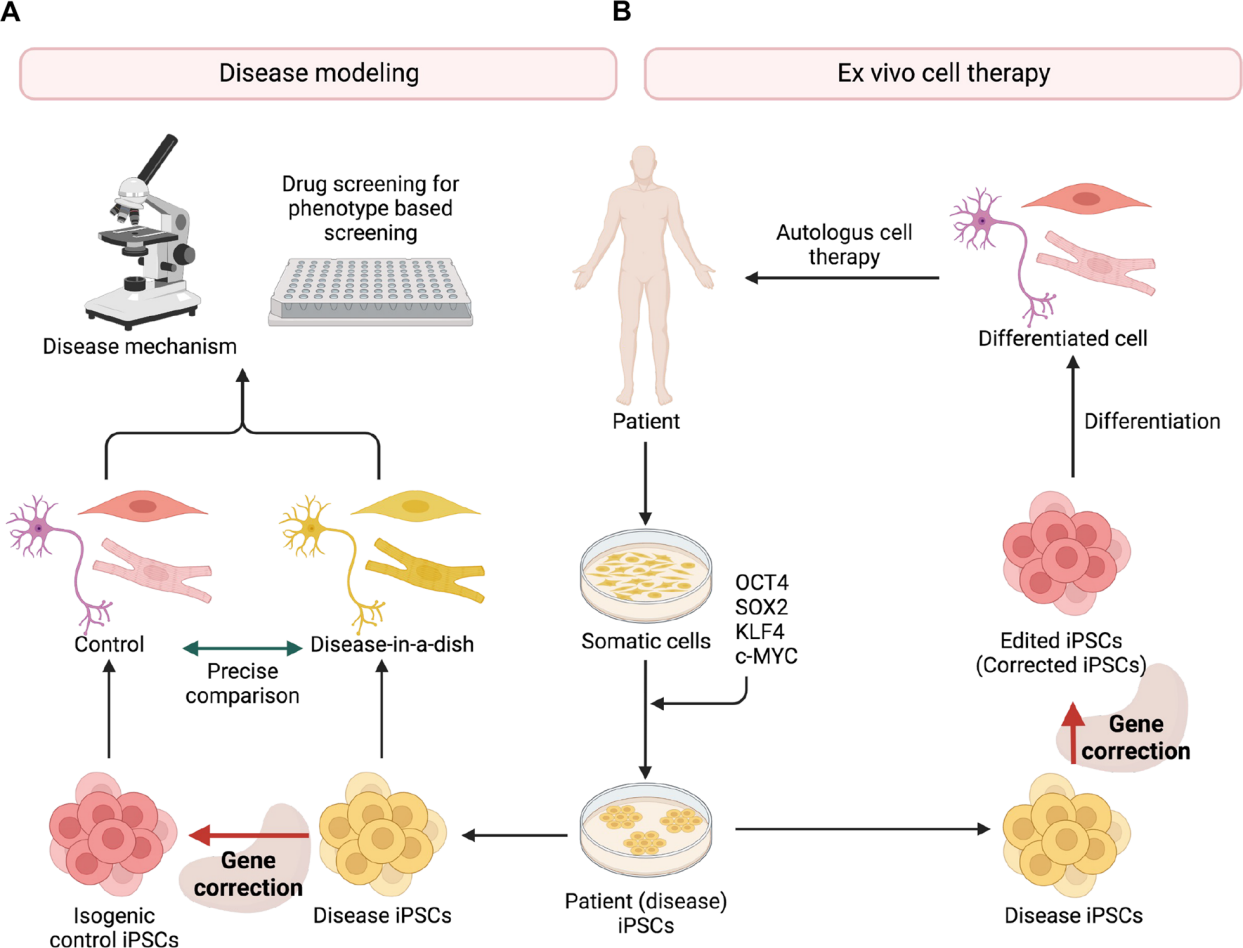
Application of patient derived iPSCs for disease modeling and cell therapy (**A**) Establishment of patient derived iPSCs (or disease iPSCs) allows the production of somatic cells with pathogenic phenotypes (i.e.*,* “Disease-in-a-dish”), which would be ultimate cell source to study the molecular mechanism to underlying disease and to screen small molecules to revert the phenotypes. Gene correction is critical to produce the isogenic control iPSCs to enable the precise comparison to avoid the variation from the different genetic background. (**B**) Autologous cell therapy from the patient with a genetic disease is achieved by gene correction of pathogenic mutations from disease iPSCs. The functionally intact somatic cells from the edited iPSCs serve as ideal cell source for reconstitution of specific organ with disease phenotype. Created with BioRender.com

The establishment of induced pluripotent stem cells (iPSCs) from human somatic cells [1] was a breakthrough not only for regenerative medicine to enable the autologous stem cell therapy but also for generating cells of any type with pathogenic phenotypes for drug discovery [2]. Thus, soon after the discovery of iPSCs, patient iPSCs harboring pathogenic mutations have been established [3] with the aims of (i) understanding the underlying mechanisms of disease and (ii) utilizing the cellular platform to assess candidate drugs based on disease phenotypes (i.e., phenotype-based drug screening; Fig. 1A) [4]. The new terminology “diseases-in-a-dish” was coined to indicate the cell-type specificity of the cells derived from patient iPSCs to reveal the pathogenic characteristics (or pathogenic phenotypes) [5]. However, the differences in cellular characteristics originating from differences in genetic backgrounds of individual patients are frequently more robust than those associated with the disease itself, which complicates the process of comparative analysis. Thus, genome editing techniques capable of specifically targeting desired sequences are essential for the establishment of isogenic pairs of disease and control human pluripotent stem cells (hPSCs) to enable “precise comparison” [6]. Furthermore, the success of the first autologous stem cell therapy utilizing cells derived from iPSCs for the Parkinson’s disease [7] opens a new chapter for autologous stem cell therapy [8]. In parallel with autologous stem cell therapy for degenerative diseases, functionally intact (i.e., devoid of mutations) cells derived from the edited forms of iPSCs initially obtained from the patients constitute a promising source to treat diverse genetic diseases through ex vivo cell therapy (Fig. 1B). Therefore, soon after their development, the efficacy and safety of new genome editing techniques have been extensively validated in hPSCs for their potential in translational applications [9–12].

## Toolbox for precise genome editing in hPSCs

Point mutations (58%) and deletions (25%) account for the majority of pathogenic variants associated with human genetic diseases [13]. Thus, various genome editing tools for the precise correction of pathogenic mutations and for the insertion of missing sequences have been developed for potential clinical applications.

### Development of programmable nucleases

**Fig. 2:**
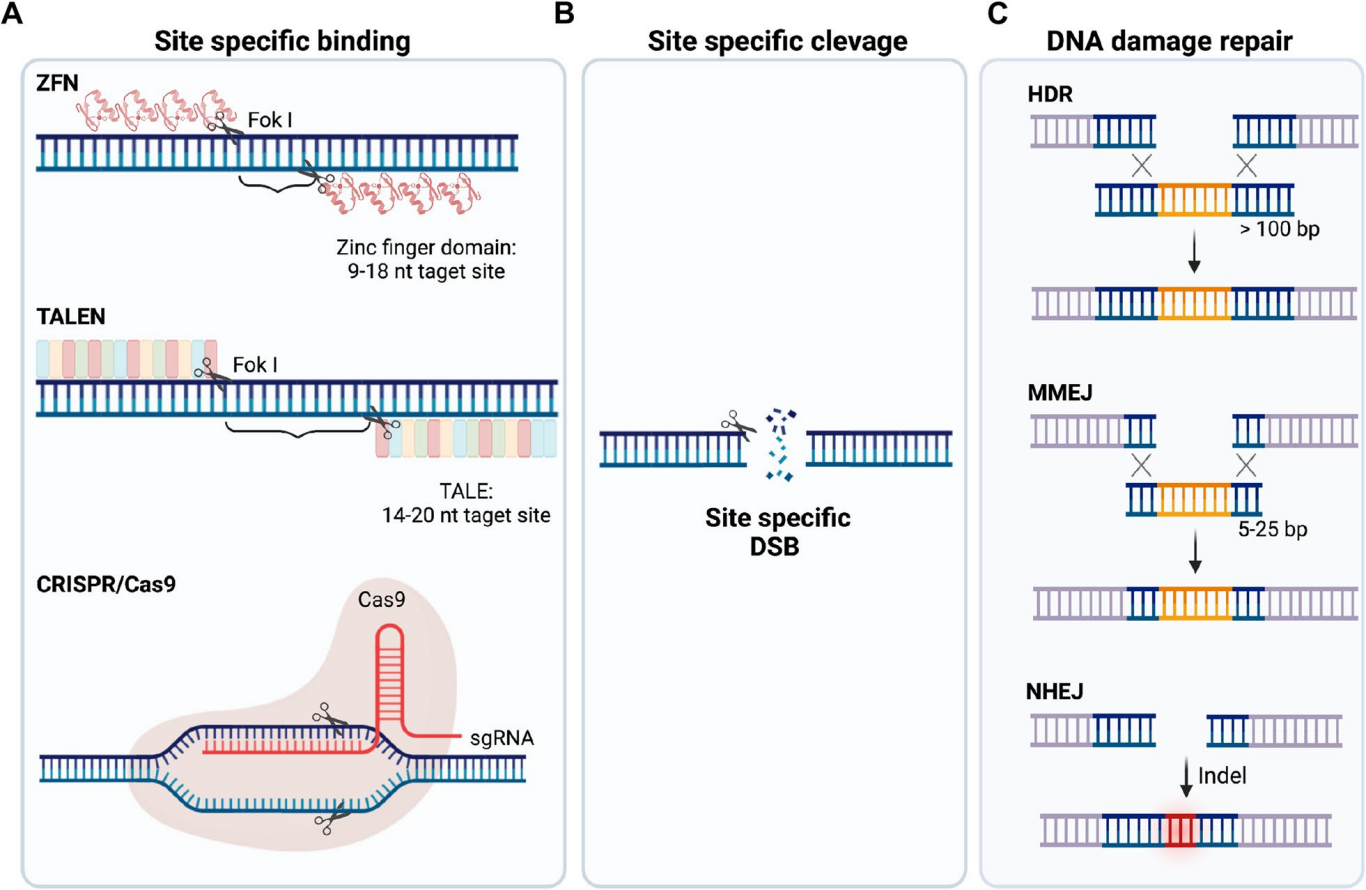
Gene editing procedure of typical programmable genome editing tools (**A**) The zinc-finger nuclease (ZFN), transcription activator-like effector nuclease (TALEN), or CRISPR/Cas9 nuclease recognize target sequence in genome (i.e.*,* “site specific binding”) by zinc-finger domain, transcription activator-like effector (TALE), or single guide-RNA (sgRNA) respectively. (**B**) The ZFN / TALEN and CRISPR/Cas9 induce “site specific cleavage” of DNA via FokI nuclease and Cas9 endonuclease respectively. (**C**) Upon DNA damage by activity of endonucleases, innate DNA damage repair system repair DNA. Site specific gene insertion from donor DNA for knock-in is achieved by homology directed repair (HDR) and micro-homology mediated end joining (MMEJ). Insertion or deletion (Indel), leading to functional knock-out occurs by non-homologous end joining (NHEJ). Created with BioRender.com

In order to manipulate genomic sequences in a programmable manner, various nucleases such as zinc finger nucleases (ZFNs) [14], transcription activator-like effector nucleases (TALENs) [15], and clustered regularly interspaced short palindromic repeats (CRISPR)/CRISPR-associated protein (Cas) system have been developed [16–19]. These programmable nuclease systems (i.e., editing tools) consist of a “DNA binding module” to guide the system to a specific DNA sequence and a “DNA-cleavage module” to cleave the target DNA sequence [20]. Upon the recruitment of “DNA-cleavage module” to the target site by the “‘DNA binding module” (Fig. 2A), site-specific cleavage occurs inducing a double strand break (DSB) through the action of “DNA-cleavage module” (Fig. 2B).

ZFN and TALENs commonly use FokI endonuclease for inducing a DSB at target sites, which is led by specific binding to target sequence of either zinc finger domain [14] or transcription activator-like effector (TALE) protein, respectively [15]. Similarly, the site-specific DNA cleavage in CRISPR/Cas system (like scissors) is conducted by single guide RNA (sgRNA) and conjugated Cas9 endonucleases [16–19]. Gene editing occurs at the site of DNA cleavage by the Cas9 endonuclease activity during the process of DNA damage repair (Fig. 2C). The desired DNA sequences from the accompanied donor DNA are inserted into the damaged DNA site [achieving knock-in (KI)] through the innate homology directed repair (HDR) or microhomology-mediated end joining (MMEJ) pathways [21, 22]. In parallel with HDR, non-homologous end joining (NHEJ) repair, an error-prone DSB repair mechanism dominantly occurring upon DSB produces random insertion or deletion (indel) mutations, leading to functional knock-out (KO) due to frame-shift (Fig. 2C). It is well-documented that 75% of DSBs are repaired by NHEJ and the remaining 25% are repaired by HR. This overall 3:1 ratio between NHEJ and HR [23] in the mammalian cells, which is altered in a cell cycle-dependent manner [24], accounts for the majority of NHEJ-associated indel mutations over HDR mediated KI by Cas9. Thus, the inevitable indel mutations for precise genome editing (base substitution or insertion) in hPSCs require the additional laborious clonal selection [21]. Alternatively, newly developed editing tools to be programmed precisely editing the desired bases (like pencil) without inducing DSBs, rather than just cutting the target DNA (like scissors), are highlighted.

### Base editors

**Fig. 3:**
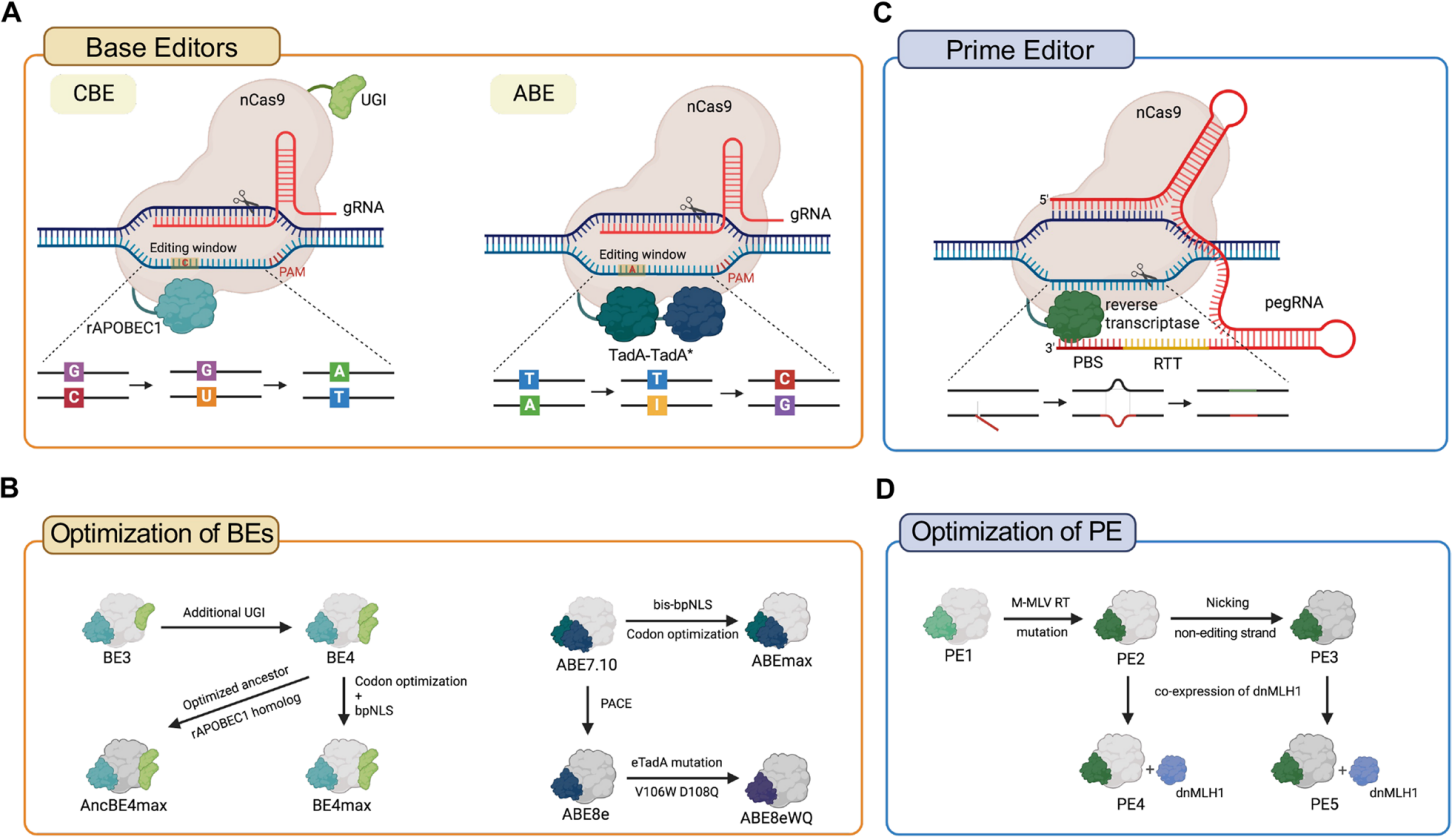
Molecular modules of BEs and PE (**A**) Base editors consist of nickase Cas9 (nCas9) and deaminase. CBE adopts rAPOBEC deaminase for cytosine deamination. For further improvement, uracil DNA glycosylase inhibitor (UGI) is conjugated. ABE adopt two deaminases (TadA-TadA*) composed of wild type TadA and engineered TadA (TadA*). (**B**) Editing efficiency and product purity of BEs are continuously improved by optimization of BEs. The original version of CBE (BE3) is further optimized to BE4, BE4max or AncBE4max with additional UGI, codon optimization and/or adoption of ancestor rAPOBEC1 homolog. The original version of ABE, ABE7.10, is optimized to ABEmax by codon optimization and adoption of bis-bpNLS. Further engineering TadA* by PACE or induction of specific mutations (e.g., V106W and D108Q) produces ABE8e and ABE8eWQ. (**C**) PE is composed of engineered reverse transcriptase (i.e.*,* M-MLV RT) linked to nCas9 and PE guide RNA (pegRNA). M-MLV RT synthesizes DNA strand containing desired edit sequences. The edit strand is inserted into the target sequence. (**D**) The original version of PE (i.e.*,* PE1) is optimized to PE2 by induction of mutation on M-MLV RT. PE3 is developed by nicking non-editing strand. Co-expression of dnMLH1 with PE2 and PE3, to further improve the efficiency produces PE4 and PE5 respectively. Created with BioRender.com

Base editors (BEs) use a deaminase linked to modified Cas proteins (unable to induce DSBs due to lack of endonuclease activity) for the site-specific base substitution [25]. Cytosine base editor (CBE) produces C:G to T:A base substitution through the action of cytosine deaminase (e.g., rat APOBEC1 [rAPOBEC1]) conjugated to nickase Cas9 (nCas9) [26]. The original version of CBE (BE3) is further optimized by adding uracil glycosylase inhibitor (UGI) resulting in BE4 for improved efficiency and product purity [27]. Additional optimization and improvement based on BE4 is continuously carried out. For example, the updated versions of CBE (BE4max and AncBE4max [28]) are produced by codon optimization or adoption of optimized ancestor rAPOBEC1 homolog (Fig. 3A). Adenine base editor (ABE) induces A:T to G:C point mutation by deaminating A via engineered adenine deaminase (e.g., TadA7.10) linked to nCas9 [29]. The original version, ABE7.10, is upgraded by the replacement of the nuclear localization sequence (NLS) with a bipartite NLS linked to both N-terminus and C-terminus (bis-bpNLS) and codon optimization (ABEmax) [28]. The adenine deaminase TadA7.10 is also improved by phage-assisted non-continuous and continuous evolution (PACE) to produce ABE8e and ABE8eWQ by introducing further point mutations in TadA8e (V106W and D108Q) [30] (Fig. 3A). In addition to transition mutations, C-to-G base substitution is achieved by C-to-G base editors (CGBE1) composed of an *E. coli*-derived uracil DNA glycosylase (eUNG) and mutant rAPOBEC1 fused to nCas9 [31] through the induction of apurinic/apyrimidinic site (AP site) by UNG activity.

### Prime editors

Unlike BEs, which can induce only certain types of point mutations (transition and C-to-G mutations), prime editors (PEs) can induce not only all 12 types of transition/transversion point mutations but also insertions and deletions without inducing DSB and requiring donor DNA [32]. PEs conduct precise genome editing by synthesizing DNA with desired mutation on the target site via PE gRNA (pegRNA) and engineered Moloney murine leukemia virous (M-MLV) reverse transcriptase (RT) [32]. After nCas9 induces DNA single strand break (SSB), primer binding site (PBS) of pegRNA binds to cleaved single strand DNA and allows RT to synthesize the DNA strand complementary to reverse transcriptase template (RTT) containing the editing information [32]. Nicking non-editing strand during prime editing (PE3) dramatically increases PE efficiency. Furthermore, co-expression of dominant negative MLH1(MutL Homolog 1) is applied to PE system (in PE4 and PE5) resulting in a significant increase in PE efficiency (Fig. 3B) [33, 34].

## Unique cellular characteristic of hPSCs affecting genome editing outcome

The maintenance of genome integrity, highly developed in human embryonic stem cells (hESCs), is one of the most distinct cellular characteristics of hESCs compared to somatic cells [35]. Thus, spontaneous mutation frequency in hESCs during in vitro culturing is 40-fold lower than those in other somatic cells [36]. This unique feature is achieved by drastic sensitivity to DNA damage stress and highly developed DNA damage repair systems in hESCs [35]. It is noteworthy that iPSCs of which most of cellular characteristics share those of hESCs [1, 37], showing similar DNA damage responses such as hypersensitivity [38] and active DNA damage repair [39, 40]. The common cellular characteristics of hESCs and iPSCs (i.e., hPSCs) upon DNA damage are well summarized in multiple review articles [35, 37, 41, 42]. As various types of DNA damage, including DSB, single strand break (SSB), or mismatch, inevitably occurs by genome editing procedure, the editing outcomes in hPSCs would not be identical to those in somatic cell lines.

### High susceptibility to DNA damage stimuli

**Fig. 4:**
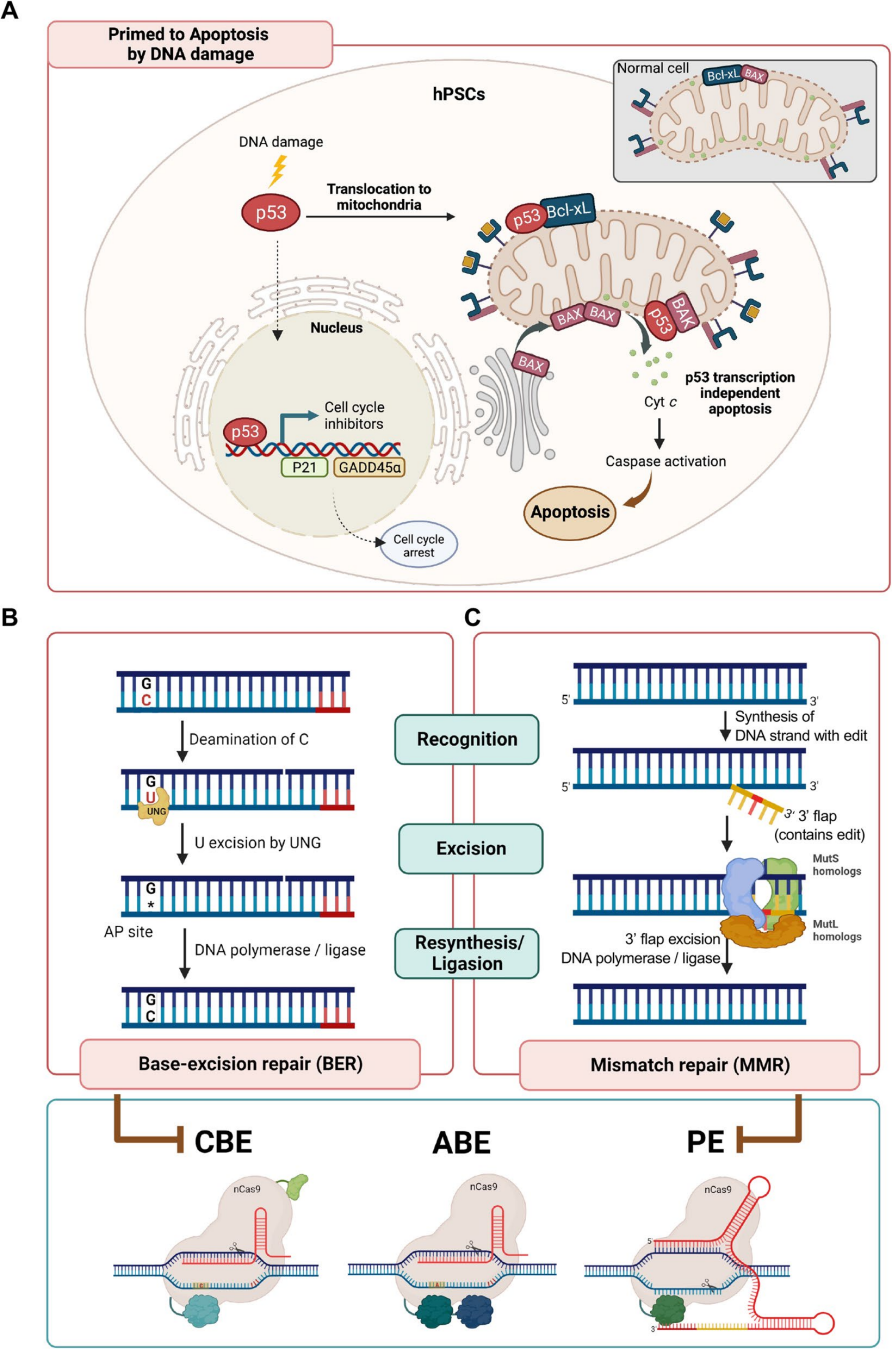
Unique cellular characteristic of hPSCs affecting genome editing outcome (**A**) hPSCs are highly susceptible to DNA damage (Primed to apoptosis). Upon DNA damage, p53 preferably translocates to mitochondria disrupting the mitochondrial membrane permeability (MMP) by direct interaction to BCL2-xL or BAK. Disrupted MMP induce cytochrome C (Cyt C) release into cytosol, which provokes mitochondrial dependent apoptosis. The transcription of cell cycle inhibitors by p53 to induce cell cycle arrest is markedly attenuated in hPSCs. (**B**) Deamination of C, producing U activates BER. U is readily recognized and removed to produce AP site by DNA glycosylase such as UNG. The high BER activity in hPSCs affects CBE outcomes. (**C**) Prime editor (PE) synthesizes DNA strand containing edit (3’ flap). The 3’ flap bound to non-editing strand is recognized by MutS and MutL homologs, major components of mismatch repair (MMR). Highly active MMR determines PE efficiency. Created with BioRender.com

A well-characterized tumor suppressor mediating diverse stress responses, p53, is readily stabilized by genotoxic stress and triggers either apoptosis or cell cycle arrest in a transcription-dependent manner [43]. Unlike somatic cells, which induce cell cycle arrest through p53-dependent gene expression of cell cycle inhibitors, hESCs tend to undergo massive cell death upon even slight genotoxic stress through the action of p53 [44]. In particular, p53 is preferably translocated into the mitochondria to prime apoptosis in hESCs [45] and iPSCs [38]. The following disruption of the mitochondrial membrane permeability (MMP) by direct interaction to BAK [46] or BCL-xL [47] to activate BAX activation [48], which leads to cytochrome C (Cyt C) release to trigger mitochondria-dependent apoptosis in hESCs upon DNA damage (i.e., p53 transcription-independent apoptosis [47]) [38, 45] (Fig. 4A). Furthermore, elevated expression of pro-apoptotic factors [38] as well as prompt translocation of active BAX, a pro-apoptotic member of BCL2 family, to mitochondria [49] accounts for the high susceptibility to DNA damage in hPSCs [50]. Accordingly, p53 activation in response to DSB induction by Cas9 endonuclease activity [51] leads to massive cell death in hPSCs, which accounts for the lower editing efficiency in hPSCs [52]. Of note, p53 activation in hPSCs also occurs as a result of nCas9 activity, which induces single strand break. Thus, editing efficiencies of BEs (both ABE and CBE) and PEs are enhanced upon genetic perturbation of *TP53* in both hESCs and iPSCs [11, 53].

### Active DNA repair systems

As the genome editing is achieved by DNA damage and consequent activity of DNA damage repair systems, the highly activated DNA damage repair pathway in hPSCs [54, 55] affects the genome editing outcomes. In particular, base excision repair (BER) targets DNA damage formed by spontaneous deamination, alkylation, or oxidation of bases [56]. These damaged bases are recognized and removed by diverse types of DNA glycosylases, including UNG, TDG, and MBD4 [57] (Fig. 4B). C-to-U deamination, the most frequent spontaneous alteration occurring in somatic cells, is a significant cause of somatic C-to-T mutations [58]. To minimize the formation of C-to-T mutations, presence of U is promptly recognized by multiple DNA glycosylases (UNG, MBD4, and TDG) to produce an AP site. Unlike UNG, which mainly recognizes G:U and A:U mismatches, TDG and MBD4 also recognize G:T mismatches [57]. Importantly, the intermediate deaminated DNA products such as U:G from C:G (by CBE) and I:T from A:T (by ABE) are recognized and removed by UNG, MBD4, TDG [57, 59, 60] and MPG [61], respectively. Recent studies have revealed that the frequency of C-to-T transition with CBE is significantly lower than that of A-to-G transition with ABE exclusively in hPSCs. Among the three typical DNA glycosylases UNG, TDG, and MBD4, which exhibit downregulated expression levels during differentiation of hPSCs, UNG has been identified as the main player to impede the editing outcome of CBE (i.e.*,* editing efficiency and product purity) [11] (Fig. 4B).

Similarly, short nucleotide sequences produced by reverse transcriptase (RT) conjugated with nCas9 in PEs (e.g., PE2 [32]) trigger mismatch repair (MMR) activation [32]. The intermediate product formed by the annellation of 3’-flap to non-editing strand and excision of the original strand (5’-flap) is recognized by three human MutS homologs (hMSH2, hMSH3, and hMSH6), initiating mismatch repair (MMR) activation (Fig. 4C). Thus, transient interference of MMR activity by inhibition of MutL homologs improves the editing outcome of PEs [33]. Accordingly, high expression levels of MSH2 and MSH6 reflecting the activity of MutSα (MSH2-MSH6 complex) and MutSβ (MSH2-MSH3 complex) in hPSCs serve as major determinants of editing outcome of PE in hPSCs [62].

## Applications of “pencil” in hPSCs

As the significance of gene editing in hPSCs is highlighted [63–65], HDR-mediated KI with Cas9 has been extensively applied to hPSCs soon after its development. The low efficiency of HDR mediated KI in hPSCs has also been improved by a number of methodologies [10, 66, 67]. As a result, Cas9 has become a standardized approach for gene perturbation or correction in hPSCs as evidenced by numerous review articles [68–70]. However, the recently developed pencil like-editing tools (i.e.*,* BEs and PEs) have not been widely utilized in hPSCs in comparison to HDR-mediated KI with Cas9. In this section, we have summarized a few examples of their usage in hPSCs (Table 1).

**Table 1:** Base substitution in hPSCs with base editing tools

Disease	Cell type	Editing tool	Mutations		Phenotype	Reference
Long QT (LQT)	hESCs, hiPSCs	ABE	L114P, R190Q	KCNQ1	Prolonged QT beating interval	Qi et al. [72]
			Y616C, Y475C	KCNH2		
GNE myopathy	hESCs, hiPSCs	ABE	I329T, I588T	GNE	Reduced sialic acid production in hPSCs and myoblasts	Park et al. [9]
		CBE	R160Q, V727M			
Recessive Dystrophic Epidermolysis Bullosa (RDEB)	Patient-derived iPSC	ABE	R185* (*non-sense mutation)	COL7A1	Deposition of C7 at the dermal–epidermal junction	Osborn et al. [74]
Duchenne muscular dystrophy (DMD)	Patient-derived iPSC	ABE	Exon 50 skipping	DMD	Restoration of dystrophin protein level in differentiated cardiomyocyte	Chemello et al. [76], Yuan et al. [75], Wang et al. [79] Eberherr et al. [77]
		PE	Exon 52 reframing			
			(GT insertion)			
		CBE	Modulating mRNA splicing			
	DMD hiPSCs established by CRISPR-Cas9 gene editing	ABE	Exon 55 skipping			
STAT3-Hyperimmuno-globulin	Patient-derived iPSC	ABE	R382W	STAT3	Restoration of STAT3 downstream signaling	
E syndrome (STAT3-HIES)						
Parkinson’s disease (PD)	Patient-derived iPSC	ABE	G2019S	LRRK2	Reduced LPRRK2 kinase activity, decreased phospho-α-synuclein expression, mitigated neurite shrinkage, apoptosis and restored impaired neurite outgrowth in differentiated dopaminergic neuron	Chang et al. [78]
	iPSCs, hESCs	PE	G2019S A30P	LRRK2 SNCA	n.a	Li et al. [80]
Dilated cardiomyopathy (DCM)	iPSCs	ABE	R634Q	RBM20	Normal distribution of RBM20 in cardiomyocytes, TTN splicing pattern, and expression of N2B	Nishiyama et al. [81]
		PE	R636S			

Disease modeling in hPSCs starts with the introduction of point mutations into normal hPSCs. Once the disease iPSCs harboring pathogenic mutations are established, the pathogenic phenotypes are determined in cell types of interest after differentiation, in comparison to the isogenic control cells. It is also noteworthy that point mutations of which pathogenicity has not been fully characterized (i.e.*,* variants of uncertain significance; VUS) could be experimentally examined by the comparison of disease models with clear pathogenic phenotypes. For example, hPSCs with point mutations occurring in patients of GNE myopathy (also known as hereditary inclusion body myopathy; HIBM), an autosomal recessive degenerative skeletal muscle disorder, were established using base editors [9]. As decreased sialic acid production, a final product of GNE (glucosamine UDP-N-acetyl-2-epimerase/N-acetylmannosamine kinase) due to loss of function mutations in epimerase or kinase domain of GNE, is closely associated with the pathogenicity of GNE myopathy, the levels of sialic acid production in each mutant hPSCs or myoblasts derived from these mutant iPSCs (including one VUS) have been used to predict the clinical significance [9].

Congenital long QT syndrome (LQTS), classified into LQT1, LQT2, and LQT3, arises from the mutations in KCQN1, KCNH2, and SCN5A, respectively [71]. A recent study has established five LQTS disease hPSC models including two LQT1, two LQT2, and one LQT3 and characterized the pathogenic phenotypes of LQTS from cardiomyocytes from hPSCs. Of note, one LQT3 model with a novel mutation identified in a Brugada syndrome (BrS) patient recapitulates BrS phenotypes at the cellular level [72]. Also, an independent protocol article has been published describing the generation of hPSCs carrying pathogenic LQTS mutations using base editors [73].

Correction of pathogenic mutations from patient derived iPSCs further strengthens the advantages of using hPSC for autologous cell therapy due to avoidance of immunological issues. Accordingly, base substitutions are performed in patient-derived iPSCs, followed by validation of restored cellular phenotypes. For example, iPSCs of patients with recessive dystrophic epidermolysis bullosa (RDEB) caused by nonsense mutations in *COL7A1* gene are edited using ABE. As nonsense mutations in *COL7A1* lead to failure of production of type VII collagen (C7), the phenotypic correction after base editing is readily examined by restoration of C7 expression not only in differentiated cell type but also in teratoma formed in mouse model [74]. Similarly, out-of-frame deletions typically occurring at exon 51 of iPSCs from Duchenne muscular dystrophy (DMD) patients are corrected using base editors. The phenotypic restoration after base correction is assessed by restoration of dystrophin protein expression in cardiomyocytes differentiated from mutation-corrected iPSCs [75]. A similar procedure is carried out in DMD iPSC model (∆Ex51 iPSCs), which is derived from a normal iPSC line. The introduction of a single nucleotide transition at the splice donor site of exon 50 induces exon skipping, and its correction restores dystrophic expression in cardiomyocytes [76]. Furthermore, prime editing is applied in ∆Ex51 iPSC-derived cardiomyocytes directly to achieve the functional recovery of cardiomyocytes [76].

The patient iPSCs from STAT3-Hyperimmunoglobulin E syndrome (HIES), a primary immunodeficiency disease due to heterozygous *STAT3* mutation, are base-edited using ABE to restore STAT3 signaling [77]. As previously described [9], base editors, especially ABE, are more efficient for base correction of leucine-rich kinase2 (LRRK2), the dominant gain-of-function mutation in Parkinson’s disease (PD), compared with HDR with no apparent indels or off-target editing [78].

## Pros and cons of BEs and PE in hPSCs

### Gene pencil rather than gene scissors

Recent studies highlight that application of Cas9 for HDR mediated KI produces large and unexpected deletions even at the chromosome level [82–84], which raises important safety concerns for its clinical applications. Importantly, isogenic pairs established by HDR-mediated KI from the patient iPSCs are later found to be hemizygous (9 out of 27 iPSCs) due to large on-target defects [85]. Similarly, up to 40% of iPSCs show large mono-allelic genomic deletions and loss-of-heterozygosity when edited with HDR-mediated KI [86]. Such large deletions extending over kilobases near the target sites result from DSB formation by Cas9 endonuclease activity [83] as the use of nCas9, which induce SSBs instead of DSBs [26, 32] significantly reduces large on-target defect [87, 88]. Hence, the use of gene editing tools such as BEs and PE based on nickase activity of nCas9 (gene pencils) is considered safer for translational applications of hPSCs as they can avoid on-target and off-target indels as well as chromosomal deletions, which are frequently observed in HDR-mediated KI [87, 88]. As a result, gene pencil would be a more suitable option for genetic manipulation in hPSCs compared to gene scissors. Additionally, it is worth mentioning that a stepwise protocol for BEs in hPSCs has been recently updated, for successful base substitution in hPSCs [89].

### Limitation of BEs and PE

**Fig. 5:**
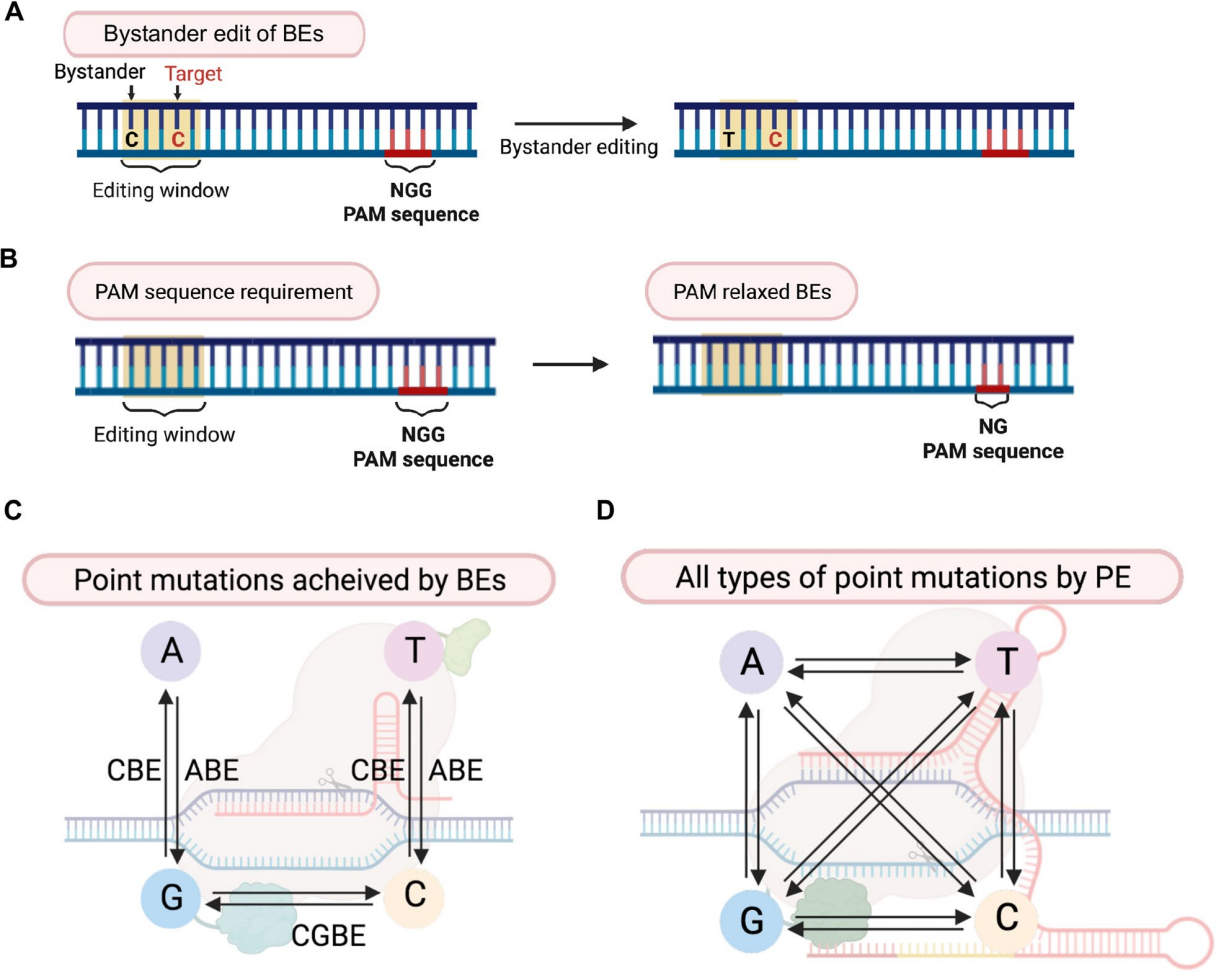
Limitation of BEs (**A**) The existence of multiple substrates in the editing windows causes unintended bystander editing. (**B**) BEs require PAM sequence (red bases) at proper distance from the target base in editing window (yellow box). The PAM-relaxed BEs (e.g., BEs with NG PAM) to extend the coverage of BEs on target mutations are developed. (**C**) Typical BEs edit only transition mutations. CGBE enables C:G to G:C base substitution. (**D**) PE edits transition and transversion point mutations. Created with BioRender.com

The presence of bystander base(s), a substrate base for deaminase but not a target base, in the editing window (or “activity window”) often produces unintended base substitution, so that laborious clonal selection after base substitution becomes necessary. Base editor variants with narrower activity windows have been developed [90]. Although BEs conduct precise genome editing without introducing DSBs, mutation scope of BEs is confined to specific types of point mutations (e.g., C:G to T:A by CBE, C:G to G:C by CGBE, and A:T to G:C by ABE) [26, 29, 31]. Furthermore, due to the requirement of the PAM sequence at the exact location of the target base, applicability of BEs to point mutations becomes limited [25]. Various versions of BEs with released PAM requirement (e.g., from NGG to NG) or near PAM-less BEs have been developed [91, 92]. PAM-relaxed version of BEs significantly increases the number of pathogenic mutations that can be targeted [9] (Fig. 5A). For example, by replacing BEs (i.e.*,* ABE and CBE with NGG as a PAM) with NG-BEs (i.e.*,* NG-ABE and NG-CBE), accessibility of pathogenic mutations associated with GNE myopathy (OMIM #605,820) extended from 15 to 38% [9]. Similarly, the coverage of mutations associated with Tay-Sachs disease (OMIM#272,800) in NG-BEs (24%) is significantly higher than that in BEs (13%) (unpublished data). Unlike the limited base substitution enabled by currently developed BEs (Fig. 5B), PEs can theoretically replace all types of point mutations as well as indel mutations [32] (Fig. 5C). Unlike HDR-mediated KI, the number of nucleotides inserted by PEs is limited to 44 base pairs [32], which would not be adequate for the targeted integration of a therapeutic gene in patient iPSCs.

The limited editing efficiency of BEs and PEs in hPSCs, caused by their unique DNA damage response characterized by p53-dependent cell death and active DNA damage repair, can be improved through temporary modulation of this response. One approach is the use of dominant negative p53 to interfere temporarily with the p53-dependent cellular response, which has been shown to enhance editing outcomes of CBE and PE in hPSCs [53]. Additionally, temporary inhibition of specific DNA damage repair pathways, such as the BER pathway for CBE with UNG depletion [11] or the MMR pathway for PE with dominant negative MLH1 expression [33], has also been found to improve efficiency in hPSCs.

## Conclusions

There have been multiple milestones in more than hundred years of stem cell research. A recent review article published in Stem Cell Reports highlighted twenty-five major discoveries in stem cell research [93], which include “nuclear transfer”, “establishment of embryonic stem cells”, “induced pluripotent stem cells”, and “organoids”. Of note, the successful autologous stem cell therapy toward junctional epidermolysis bullosa (JEB) patients using epidermal stem cells after gene correction (retroviral transduction of *LAMB3*) [94] needs to be highlighted. The current genome editing technology is capable of directly correcting the pathogenic mutations while avoiding the introduction of a transgene, providing safer therapeutic stem cell sources. Thus, when “hPSCs meet genome editing” [65], further milestones not limited to stem cell research would be expected in future.

## Data Availability

Not applicable.

## Abbreviations

hPSCs: Human pluripotent stem cells
iPSCs: Induce pluripotent stem cells
ZFNs: Zinc finger nucleases
TALEN: Transcription activator-like effector nucleases
CRISPR: Clustered regularly interspaced short palindromic repeats
Cas: CRISPR-associated protein
DSB: DNA double strand break
TALE: Transcription activator-like effector
sgRNA: Single guide RNA
KI: Knock-in
HDR: Homology directed repair
MMEJ: Microhomology-mediated end joining
NHEJ: Non-homologous end joining
Indel: Insertion or deletion
KO: Knock-out
BEs: Base editors
CBE: Cytosine base editor
UGI: Uracil glycosylase inhibitor
ABE: Adenine base editor
NLS: Nuclear localization sequence
PACE: Phage-assisted non-continuous and continuous evolution
AP site: Apurinic/apyrimidinic site
UNG: Uracil DNA glycosylase
PEs: Prime editors
pegRNA: PE gRNA
RT: Reverse transcriptase
SSB: DNA single strand break
PBS: Primer binding site
RTT: Reverse transcriptase template
MMP: Mitochondrial membrane permeability
Cyt C: Cytochrome C
MMR: Mismatch repair
VUS: Variants of uncertain significance
HIBM: Hereditary inclusion body myopathy
LQTS: Long QT syndrome
BrS: Brugada syndrome
RDEB: Recessive dystrophic epidermolysis bullosa
C7: Type VII collagen
DMD: Duchenne muscular dystrophy
HIES: STAT3-Hyperimmunoglobulin E syndrome
LRRK2: Leucine-rich kinase2
PD: Parkinson’s disease
PAM: Protospacer adjacent motif
